# Antiarrhythmic drug therapy and catheter ablation in patients with paroxysmal or persistent atrial fibrillation: a systematic review and meta-analysis

**DOI:** 10.1186/s12872-024-03983-z

**Published:** 2024-06-25

**Authors:** Subhash Chander, Roopa Kumari, Sindhu Luhana, Sheena Shiwlani, Om Parkash, FNU Sorath, Hong Yu Wang, Sam Tan, Zubair Rahaman, Yaqub Nadeem Mohammed, Abhi Chand Lohana, FNU Sakshi, Esha Vaish, FNU Sadarat

**Affiliations:** 1 Department of Medicine, Icahn School of Medicine, Mount Sinai Beth Israel Hospital, New York City, NY USA; 2 Department of Medicine, Montefiore Medical Centre, Wakefield, NY USA; 3 Department of Medicine, University at Buffalo, Buffalo, NY USA; 4 Department of Anesthesiology, Dow University Health Sciences, Karachi, Pakistan; 5 Department of Medicine, Western Michigan University, Kalamazoo, WV USA; 6 Department of Medicine, WVU, Camden Clark Medical Centre, Parkersburg, WV USA; 7 Department of Medicine, Piedmont Augusta Hospital, Augusta, GA USA

**Keywords:** Atrial fibrillation, Catheter ablation, Antiarrhythmic agents, Systematic review

## Abstract

**Background:**

Catheter ablation and antiarrhythmic drug therapy are utilized for rhythm control in atrial fibrillation (AF), but their comparative effectiveness, especially with contemporary treatment modalities, remains undefined. We conducted a systematic review and meta-analysis contrasting current ablation techniques against antiarrhythmic medications for AF.

**Methods:**

We searched PubMed, SCOPUS, Cochrane CENTRAL, and Web of Science until November 2023 for randomized trials comparing AF catheter ablation with antiarrhythmics, against antiarrhythmic drug therapy alone, reporting outcomes for > 6 months. Four investigators extracted data and appraised risk of bias (ROB) with ROB 2 tool. Meta-analyses estimated pooled efficacy and safety outcomes using R software.

**Results:**

Twelve trials (*n* = 3977) met the inclusion criteria. Catheter ablation was associated with lower AF recurrence (relative risk (RR) = 0.44, 95%CI (0.33, 0.59), *P* ˂ 0.0001) and hospitalizations (RR = 0.44, 95%CI (0.23, 0.82), *P* = 0.009) than antiarrhythmic medications. Catheter ablation also improved the physical quality of life component score (assessed by a 36-item Short Form survey) by 7.61 points (95%CI -0.70-15.92, *P* = 0.07); but, due to high heterogeneity, it was not statistically significant. Ablation was significantly associated with higher procedural-related complications [RR = 15.70, 95%CI (4.53, 54.38), *P* < 0.0001] and cardiac tamponade [RR = 9.22, 95%CI (2.16, 39.40), *P* = 0.0027]. All-cause mortality was similar between the two groups.

**Conclusions:**

For symptomatic AF, upfront catheter ablation reduces arrhythmia and hospitalizations better than continued medical therapy alone, albeit with moderately more adverse events. Careful patient selection and risk-benefit assessment are warranted regarding the timing of ablation.

**Supplementary Information:**

The online version contains supplementary material available at 10.1186/s12872-024-03983-z.

## Introduction

Atrial fibrillation (AF) is the most common sustained cardiac arrhythmia, estimated to affect over 37 million people worldwide, with an increasing prevalence with age [1]. AF confers significant risk for stroke, heart failure, and cardiovascular mortality, making treatment strategies aimed at controlling for AF to lower symptoms and prevent these complications a major public health priority [2, 3].

AF occurs in approximately 1–2% of younger adults, increasing to over 10% of those over 80 years old [1, 2, 4]. It often first presents with symptoms like palpitations, chest pain, fatigue, dizziness, and shortness of breath; however asymptomatic AF may exist in as many as 30% of those affected [5]. Long-standing persistent AF carries a substantial risk of tachycardia-mediated cardiomyopathy [6].

Antiarrhythmic drugs (AADs) work through various mechanisms to prevent AF recurrence but are limited by modest efficacy and risk for ventricular proarrhythmia and extracardiac toxicity [7]. Amiodarone and dofetilide are often more effective but have substantial non-cardiac adverse effects [8]. Guidelines recommend tailored AAD choice based on the presence of structural heart disease and other patient factors [9].

Catheter ablation involves using radiofrequency energy delivered to strategic locations in the left atrium to electrically isolate and ablate arrhythmogenic foci [10]. This technique aims to prevent AF triggers and maintenance [10]. Technological advancements have improved ablation success. Complications like bleeding, vascular damage and stroke remain a concern with ablation procedures [11].

Prior studies and meta-analyses suggest catheter ablation may afford greater freedom from AF recurrence compared with AADs in paroxysmal AF, but its role in persistent AF remains less defined [12, 13]. However, few studies directly compare contemporary ablation approaches to next-generation AADs [14].

Both modalities confer specific benefits and adverse effects important in treatment considerations for a given AF patient. Catheter ablation plays an important early role in management of symptomatic paroxysmal AF refractory to a single AAD. For persistent AF, decisions are more complex with both options having relatively lower efficacy [15].

In this study, we aim to systematically review randomized head-to-head trial evidence comparing outcomes of catheter ablation against antiarrhythmic drug therapy for treatment of symptomatic paroxysmal or persistent AF, providing a quantitative comparison of their efficacy and safety which can further guide clinical decision-making.

## Methods

Cochrane Handbook for Systematic Reviews and Interventions [16] and Preferred Reporting Items for Systematic Reviews and Meta-Analyses (PRISMA) standards [17] were adhered to throughout this meta-analysis. Additionally, this systematic review and meta-analysis is registered with PROSPERO international prospective register of systematic reviews under ID of CRD42023486487 on Dec 12, 2023.

### Search strategy and selection criteria

We systematically searched PubMed, SCOPUS, Cochrane Library, and Web Of Science (WOS) from database inception to November 2023. No filters were applied. The search strategy included a combination of controlled vocabulary terms and free text words for the key concepts of atrial fibrillation, antiarrhythmic medications, and catheter ablation. We also hand-searched reference lists of relevant review articles to identify additional eligible trials.

We included randomized controlled trials (RCTs) comparing antiarrhythmic drugs (AADs) versus catheter ablation, either alone or combined with AADs during the blanking period, for adult patients (≥ 18 years) with paroxysmal or persistent atrial fibrillation. Trials had to report at least one of the prespecified efficacy or safety outcomes at a minimum follow-up duration of 6 months. We excluded non-randomized studies, observational studies, case series, case reports, editorials, and conference abstracts without subsequent full publication.

### Study selection and data extraction

Two investigators independently screened all retrieved titles/abstracts and potentially eligible full-text articles, determined final study inclusion, and extracted relevant data using a standardized and piloted data extraction form. Extracted information included: study characteristics (author, year, country, AF type), patient characteristics (age, gender, ), lengths of follow-up, and results for each study. Any discrepancies during study screening and data extraction were resolved via discussion and consensus between the two reviewers, consulting a third reviewer for persistent disagreements if needed.

### Outcomes

The prespecified primary efficacy outcome was arrythmia recurrence. Secondary outcomes included all-cause mortality, cardiovascular hospitalizations, change in quality-of-life scores from baseline (assessed using validated instruments such as the 36-item Short Form survey [SF-36], and adverse events, including any adverse events (AEs), procedure-related AEs, stroke, vascular access complication, cardiac tamponade, pericardial effusion, and pulmonary-vein stenosis.

### Quality assessment

The Risk of Bias (ROB) tool, version 2, was used to assess the bias of the studies used into this meta-analysis [18]. The tool evaluates five domains: bias caused by the randomization technique, bias caused by variations from planned interventions, bias caused by missing outcome data, bias in outcome assessment, and bias in the selection of the reported result. For each domain, the risk of bias was rated as low, moderate, or high. Using ROB 2, two reviewers independently evaluated each research’s bias risk. Any differences were worked out via debate and consensus.

### Data synthesis and analysis

All analyses were performed using RStudio version 2023.12.0 + 369 using the “meta” package. For outcomes reported by at least two RCTs, pooled effect estimates were calculated using fixed effect model or random-effects meta-analysis models to account for between-study heterogeneity. Dichotomous data were expressed as risk ratios (RR) and continuous data as mean differences (MD), both with 95% confidence intervals (CI). Statistical heterogeneity was assessed with the P static. If substantial heterogeneity (*P* > 0.1) existed, the random effect model and leave one test were used and leave one test was conducted using open meta-analyst software.

Subgroup analysis was carried out for the primary outcome based on the type of AF: paroxysmal, persistent, or studies combining both types. A subgroup analysis was also performed based on the period of follow-up: 6–12 months or > 12 months. A meta-regression was performed to provide further confirmation of the relationship between the pooled estimate and the duration of follow-up.

## Results

**Fig. 1:**
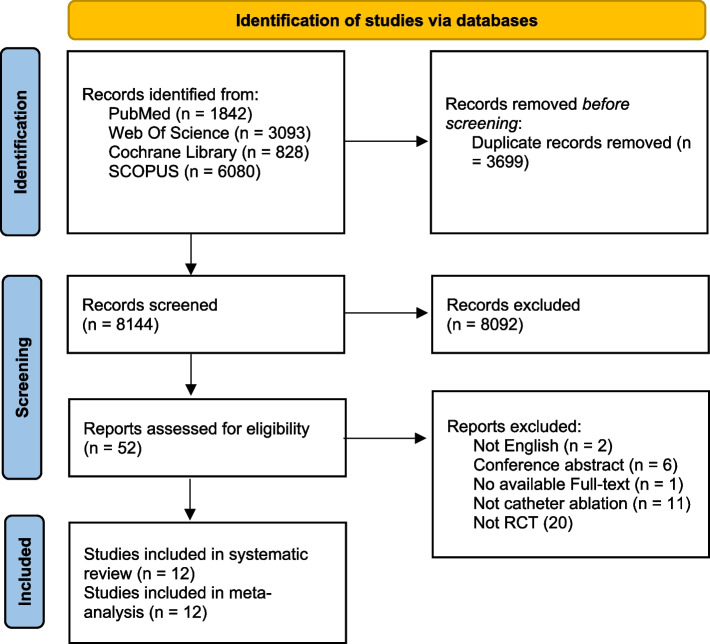
PRISMA Flow Chart

A comprehensive literature search was conducted using PubMed, Web of Science, Cochrane Library, and SCOPUS databases to identify relevant studies comparing catheter ablation to antiarrhythmic drug therapy for atrial fibrillation. The search yielded 1842 records from PubMed, 3093 from Web of Science, 828 from Cochrane Library, and 6080 from SCOPUS. After removing 3699 duplicate records, 8144 records were screened by title and abstract. Of these, 8092 records were excluded according to predefined criteria. The remaining 52 reports underwent full-text screening, after which 40 were excluded. A total of 20 randomized controlled trials ultimately met the criteria for inclusion in the systematic review, with all 12 providing sufficient data for the meta-analysis. Fig. 1.

### Summary and baseline characteristics of the included studies

Twelve randomized controlled trials comparing catheter ablation to antiarrhythmic drug therapy for atrial fibrillation were included. These trials enrolled patients with paroxysmal (7 trials), persistent (4 trials) or chronic (1 trial) AF. Follow-up duration ranged from 9 months to 4 years. Most trials were multi-national in scope. Table 1.

**Table 1:** Summary of the included studies

Study ID	NCT	Site	Type of AF	Population	Follow-up	Conclusion	Cardiac rhythm measurement	Endpoint definition	Energy type	Intervention group	Control group
**Biase et al. 2016 **[19]	NCT00729911	Multi-national	Persistent AF	Patients with congestive heart failure and an implanted device with persistent AF.	Two years	The study reported that for patients with heart failure and persistent AF, catheter ablation was superior to amiodarone in achieving long-term relieving of AF and reducing unplanned hospitalizations and mortality.	Remote monitoring with implanted devices and/or with device inspection at 3-, 6-, 12- and 24-months follow-up.	Recurrence of AF was defined as AF that lasted at least 30 s	Radiofrequency	Ablation + AADs for the first 3 months	Amiodarone (200 mg/d)
**Forleo et al. 2009 **[23]	NA	Italy	Paroxysmal [29] and persistent (41)	Type 2 diabetic patients with symptomatic paroxysmal or persistent AF for ≥ 6 months refractory to ≥ 1 class 1–3 AADs	One year	For those with type 2 diabetes, AF catheter ablation offered better outcomes than drug therapy, proving feasible, effective, and low-risk.	Holter ECG at 1, 3, and every 3 months thereafter or in case of occurrence of any clinical symptom.	AF recurrence was defined as any electrocardiographically confirmed episode of AF or atypical atrial flutter lasting > 30 s	Radiofrequency	Ablation + AADs for the first 3 months	Either as single drug or combination of oral flecainide (100 mg/12 hours), oral propafenone (150–300 mg/TID), oral sotalol at an initial dose of 80 mg/TID, and oral amiodarone (200 mg/d).
**Jais et al. 2008 **[26]	NCT00540787	USA and France	Paroxysmal AF	Patients with paroxysmal AF resistant to at least 1 antiarrhythmic drug.	One year	The study revealed that catheter ablation was more effective in sustaining sinus rhythm and enhancing symptoms, exercise capacity, and quality of life compared to the use of antiarrhythmic medications.	12-lead ECG and 24-hour Holter at baseline and 3, 6, and 12 months	Episodes qualified as AF if they lasted at least 3 min and were documented by ECG or reported by the patient as AF, even in the absence of ECG	Radiofrequency	Ablation	Amiodarone (200 mg/d), quinidine, disopyramide, flecainide, propafenone, cibenzoline, dofetilide, and sotalol (either alone or in combination)
**Kuck et al. 2021 **[25]	NCT01570361	Multi-national	Paroxysmal AF	Patients with paroxysmal AF for more than two years and more than two episodes over the last six months, resistant to at least 1 antiarrhythmic drug.	Three years	Radiofrequency ablation was superior to AADs in delaying the progression from paroxysmal to persistent AF.	ECG were conducted at 3 and 6 months, and then at yearly intervals for 3 years. Weekly transtelephonic monitoring (TTM) began at 3 months, transitioning to monthly monitoring after 9 months until the 3-year visit, or whenever subjects experienced arrhythmic symptoms	Recurrence of AF was defined as AF that lasted at least 30 s for > 7 consecutive days or requiring termination by cardioversion after 48 h	Radiofrequency	Ablation	AADs according to current guidelines at the investigators’ discretion.
**Mont et al. 2014 **[21]	NCT00863213	Spain	Persistent AF	Patients with persistent AF, requiring electrical or pharmacological cardioversion and refractory to at least one class I or class III antiarrhythmic drug.	One year	Catheter ablation was superior to medical therapy for the maintenance of sinus rhythm in patients with persistent AF at 12-month follow-up	12-lead ECG at 1, 3, 6, and 12 months and 24-h Holter monitor was performed at 6 and 12 months.	Recurrence of AF was defined as AF that lasted at least 30 s	Radiofrequency	Ablation + AADs for the first 3 months	Class III drugs (amiodarone) were recommended for patients with structural cardiomyopathy and class Ic (flecainide) plus diltiazem or b-blockers otherwise
**Morillo et al. 2014 **[20]	NCT00392054	Multi-national	Paroxysmal AF	Patients with recurrent paroxysmal AF lasting over 30 s, at least one documented episode 6 months prior, and no prior antiarrhythmic drug treatment.	Two years	For those with untreated paroxysmal AF, radiofrequency ablation resulted in fewer recurring atrial tachyarrhythmias at 2 years compared to antiarrhythmic drugs, but both groups still experienced frequent recurrence.	Continuous remote monitoring (Using transtelephonic monitor system, patients were required to record and transmit symptomatic episodes of possible AF every week, and biweekly on Fridays throughout the follow-up period, regardless of symptoms)	Recurrence of arrythmia is arrythmia lasting more than 30 s documented by ECG or transtelephonic monitor	Radiofrequency	Ablation + AADs for the first 3 months	Aِntiarrhythmic drugs was left to the discretion of the investigator, and dosages were based on guidelines
**Nielsen et al. 2012 **[28]	NCT00133211	Denmark	Paroxysmal AF	Patient experienced at least two symptomatic episodes of atrial fibrillation within the past six months, with no episode lasting more than seven days.	Two years	Comparing radiofrequency ablation to antiarrhythmic drugs as first-line therapy for paroxysmal AF, they found no significant difference in overall AF burden over 2 years.	7-day Holter-monitor recording at 3, 6, 12, 18, and 24 months. Patients were instructed to report palpitations or other symptoms to the study center between follow-up visits.	AF was defined as AF that lasted at least 1 min	Radiofrequency	Ablation + AADs for the first three months	Class IC agents such as flecainide (200 mg/day) or propafenone (600 mg/day). If contraindicated, Class III agents like amiodarone (200 mg/day) or sotalol (160 mg/day) are administered. No combination between class IC and class III was allowed
**Oral et al. 2006 **[30]	NA	Italy	Persistent AF	A patient experienced persistent atrial fibrillation for over six months without any intervening sinus rhythm episodes	One year	Patients with chronic atrial fibrillation could sustain sinus rhythm long-term through pulmonary-vein ablation, regardless of antiarrhythmic drugs or cardioversion effects.	Continuous remote monitoring (Participants were monitored using LifeWatch, recording their heart rate at least five days a week for three minutes and whenever they had symptoms of AF)	NA	Radiofrequency	Ablation + Amiodarone, 200 mg/day, orally for three months	Amiodarone, 200 mg/day, orally for three months + transthoracic cardioversions
**Packer et al. 2019 **[22]	NCT00911508	Multi-national	Paroxysmal (42.9%) and persistent (47.2%)	Symptomatic patients with AF with 1 or more risk factors for stroke (hypertension, heart failure, history of stroke, diabetes, or other heart problems), with two or more episodes of paroxysmal AF or one episode of persistent AF within the past six months.	Four years	In patients with AF, the approach of catheter ablation didn’t notably decrease the main combined outcome of death, disabling stroke, severe bleeding, or cardiac arrest when compared to medical therapy.	ECG event recorder to document symptomatic events, quarterly 24-hour autodetect, full-disclosure, real-time recordings and 96-hour Holter recordings every six months, regardless of symptoms.	Recurrence of AF was defined as AF that lasted at least 30 s	NA	Ablation	AAD
**Pappone et al. 2006 **[27]	NCT00340314	Italy	Paroxysmal AF	Patients with paroxysmal AF who have already failed AADs.	One year	CA was more successful than AAD for the prevention of atrial fibrillation with less complications.	12-lead electrocardiogram (ECG) and 48-h Holter monitoring at 3-, 6-, and 12-months visits and Continuous remote monitoring (Participants were monitored using LifeWatch were asked to record their rhythm 1 to 3 times daily and whenever they experienced symptoms suggestive of AF)	Recurrence of AF was defined as AF that lasted at least 30 s	Radiofrequency	Ablation + AADs for 6 weeks	Amiodarone (200 mg/day), flecainide (up to 300 mg/day), or sotalol (up to 320 mg/day), (either as single drugs or in combination) at the maximum tolerable doses
**Wazni et al. 2005 **[29]	NA	Italy and Germany	Paroxysmal AF	Patients had experienced monthly symptomatic AF episodes for at least 3 months.	One year	Pulmonary vein isolation was a viable initial option.	Event recorder monitoring was obtained after the 3-month period for patients with recurrences of symptoms and 24-hour Holter recording before discharge, as well as 3, 6, and 12 months after enrollment. Patients were also phoned once a month by phone.	Symptomatic or asymptomatic AF lasting more than 15 s during Holter or event monitoring	Radiofrequency	Ablation	Oral flecainide (100–150 mg) twice daily, propafenone (225–300 mg) 3 times daily, and sotalol (120–160 mg) twice daily.
**Wilber et al. 2010 **[24]	NCT00116428	Multi-national	Paroxysmal AF	Patients with at least three symptomatic AF episodes within 6 months before randomization and not responding to at least one antiarrhythmic drug are eligible.	9 months	In individuals with paroxysmal AF unresponsive to at least one antiarrhythmic drug, catheter ablation led to an extended duration until treatment failure compared to AADs over the 9-month follow-up period.	ECG were obtained at follow-up visits, and transtelephonic monitoring was performed for 9 months. Patients were required to transmit symptomatic cardiac episodes weekly, then monthly till the final visit. Holter was done at baseline and final visits.	NA	Radiofrequency	Ablation	Dofetilide, flecainide, propafenone, sotalol, or quinidine

*AF* Atrial Fibrillation, *NCT* ClinicalTrials.gov Identifier (an alphanumeric code assigned to clinical trials), *AADs* Antiarrhythmic Drugs, *NA* not available

Baseline characteristics were well-balanced between the catheter ablation and antiarrhythmic drug arms within each included trial. The mean patient age ranged from 53 to 68 years. Most trials had 60–80% male participants. The mean duration of AF history before enrollment was 1–8 years where reported. The rate of hypertension was 40–90%. Left ventricular ejection fraction averaged between 52 and 62% across trials. Table 2.

**Table 2:** Baseline characteristics of the included studies

Study ID	Study arms	Sample	Age, years, M (SD)	Sex, male (%)	AF duration, months, M (SD)	Hypertension, *n* (%)	LVEF, %, M (SD)
**Biase et al. 2016 **[19]	Catheter ablation	102	62 (﻿10)	77 (75%)	8.6 (3.2)	46 (45%)	29 (5)
	AADs	101	60 (11)	74 (73%)	8.4 (4.1)	48 (48%)	30 (8)
**Forleo et al. 2009 **[23]	Catheter ablation	35	63.2 (8.6)	20 (57.1%)	41 (18–66)*	22 (62.9%)	54.6 (7)
	AADs	35	64.8 (6.5)	23 (65.7%)	36 (17–55)*	24 (68.6%)	52.6 (8.6)
**Jais et al. 2008 **[26]	Catheter ablation	53	49.7 (10.7)	45 (84.9%)	-	11 (21.6%)	63.1 (11)
	AADs	59	52.4 (11.4)	49 (83.1%)	-	18 (30.5%)	65.6 (7.2)
**Kuck et al. 2021 **[25]	Catheter ablation	128	67.8 (4.8)	54 (42.2%)	51.2 (19–625)**	120 (93.8%)	61.8 (5.8)
	AADs	127	67.6 (4.6)	53 (41.7%)	49.8 (25–366)**	123 (96.9%)	62.3 (5.2)
**Mont et al. 2014 **[21]	Catheter ablation	98	55 (9)	76 (77.5%)	-	46 (46.9%)	61.1 (8.8)
	AADs	48	55 (9)	37 (77%)	-	19 (39.5%)	60.8 (9.7)
**Morillo et al. 2014 **[20]	Catheter ablation	66	56.3 (9.3)	51 (77.3%)	-	28 (42.4%)	61.4 (4.8)
	AADs	61	54.3 (11.7)	45 (73.8%)	-	25 (41%)	60.8 (7.0)
**Nielsen et al. 2012 **[28]	Catheter ablation	146	56 (9)	100 (68%)	-	43 (29%)	-
	AADs	148	54 (10)	106 (72%)	-	53 (36%)	-
**Oral et al. 2006 **[30]	Catheter ablation	77	55 (9)	67 (87%)	60 (48)	-	55 (7)
	AADs	69	58 (8)	67 (94%)	48 (48)	-	56 (7)
**Packer et al. 2019 **[22]	Catheter ablation	1108	68 (62–72)*	695 (62.7%)	-	876 (79.1%)	-
	AADs	1096	67 (62–72)*	690 (63%)	-	900 (82.2%)	-
**Pappone et al. 2006 **[27]	Catheter ablation	99	55 (10)	69 (70%)	72 (48)	55 (56%)	60 (8)
	AADs	99	57 (10)	64 (65%)	72 (72)	56 (57%)	61 (6)
**Wazni et al. 2005 **[29]	Catheter ablation	32	53 (8)	-	-	-	53 (5)
	AADs	35	54 (8)	-	-	-	54 (6)
**Wilber et al. 2010 **[24]	Catheter ablation	106	55.5 (53.7–57.3)*	73 (68.9%)	5.4 (4.3–6.5)*	51 (48.6%)	62.3 (60.4–64.3)*
	AADs	61	56.1 (52.9–59.4)*	38 (62%)	6.2 (4.6–7.9)*	30 (50%)	62.7 (60.7–64.7)*

*AF* Atrial Fibrillation, *AADs* Antiarrhythmic Drugs, *n (%)* Number (Percentage), *LVEF* Left Ventricular Ejection Fraction, *M* mean, *SD* standard deviation

*data presented as median and interquartile ranges, **data presented as median and ranges

### Quality assessment

**Fig. 2:**
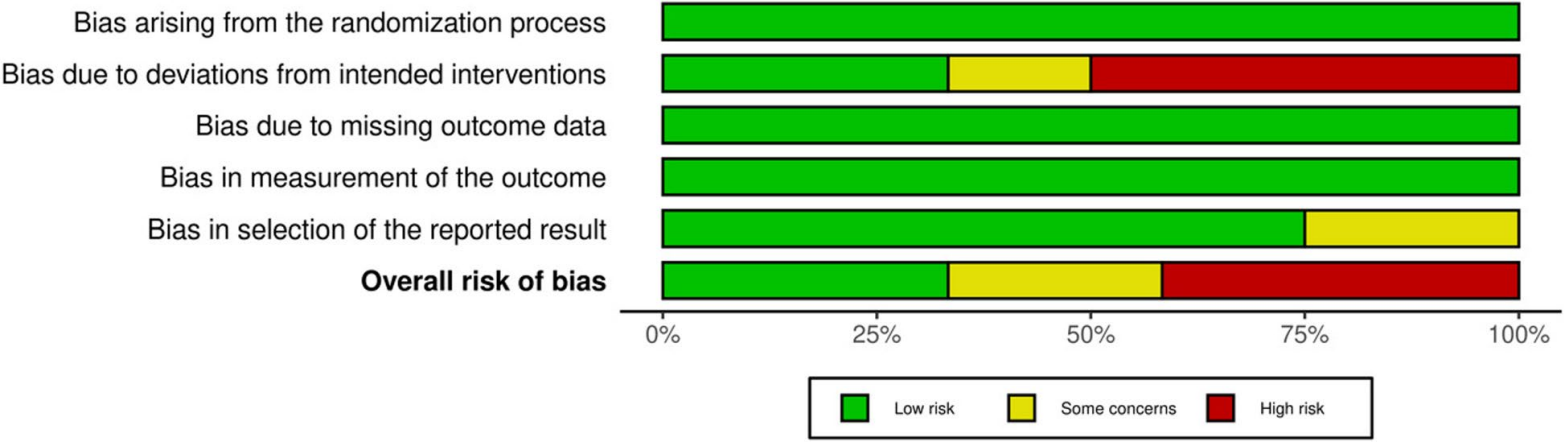
Risk of bias as a percentage

**Fig. 3:**
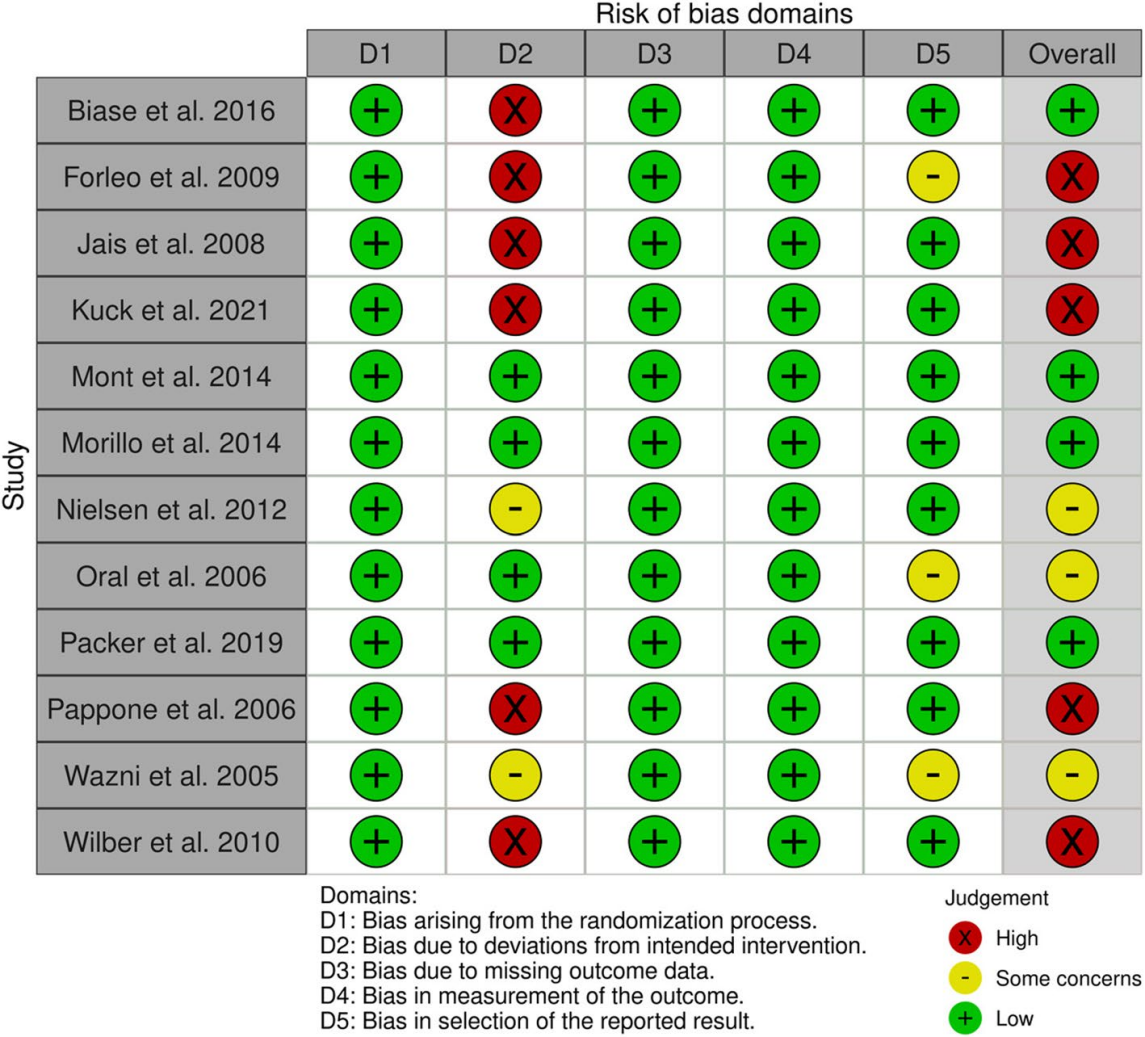
Risk of bias per protocol for individual studies

Four studies demonstrated consistent low-risk ratings across all domains, suggesting a high level of confidence in its reliability [19–22]. Conversely, five studies exhibited a high risk of bias in the deviation from intended intervention and the overall risk was high [23–27]. Finally, the rest three studies were judged as having overall some concerns due to few details about blinding and protocol registration [28–30]. Figs. 2 and 3.

### Outcomes

#### Primary outcomes

##### Recurrent atrial arrhythmia

**Fig. 4:**
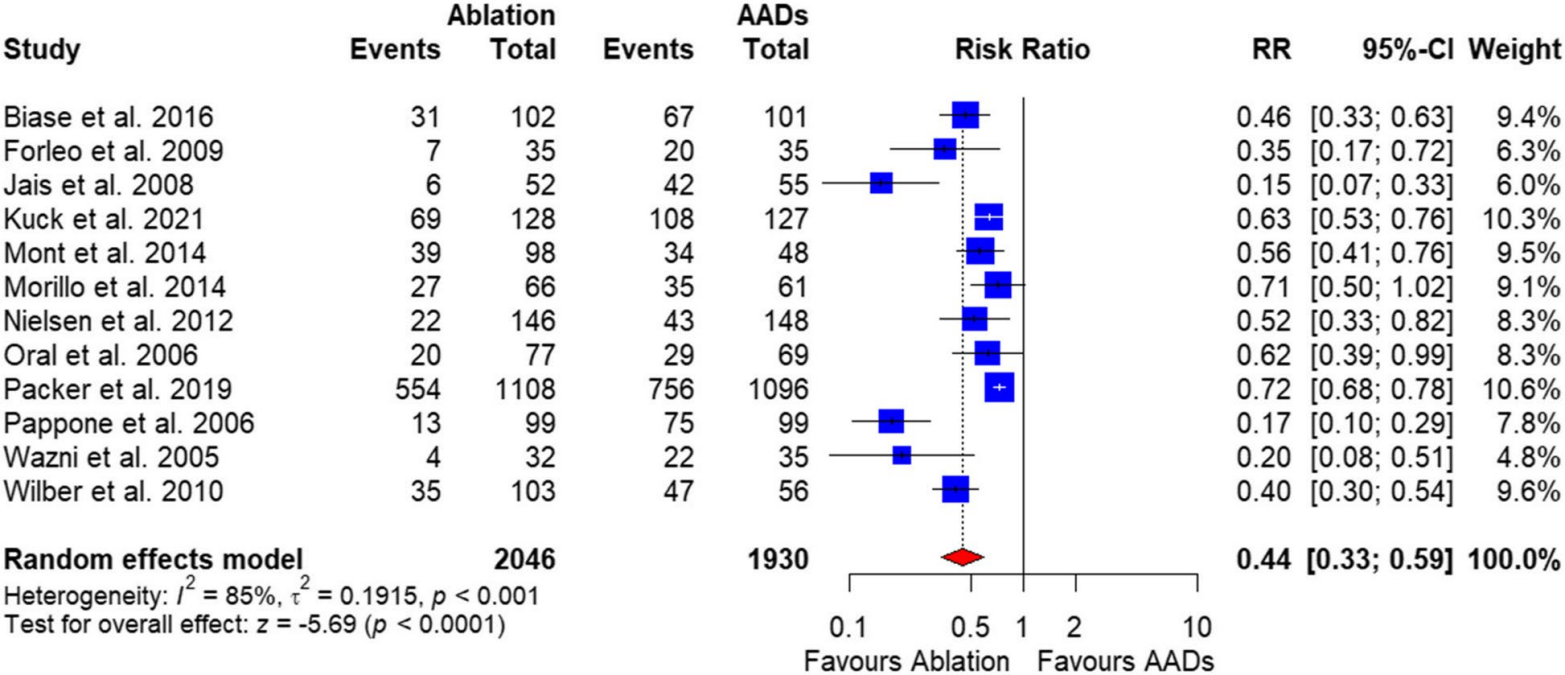
Forest plot of recurrent arrythmias

The pooled analysis of the 12 included studies with a total of 3977 patients showed a significantly lower AF recurrence rate in the catheter ablation group compared with AADs [RR = 0.44, 95%CI (0.33, 0.59), *P* ˂ 0.00001] (Fig. 4**).** The data were heterogenous (*P* ˂ 0.001, I^2^ = 85%), and this heterogeneity could not be resolved (Supplementary Fig. 1).

#### Secondary outcomes

##### Rate of hospitalization

**Fig. 5:**
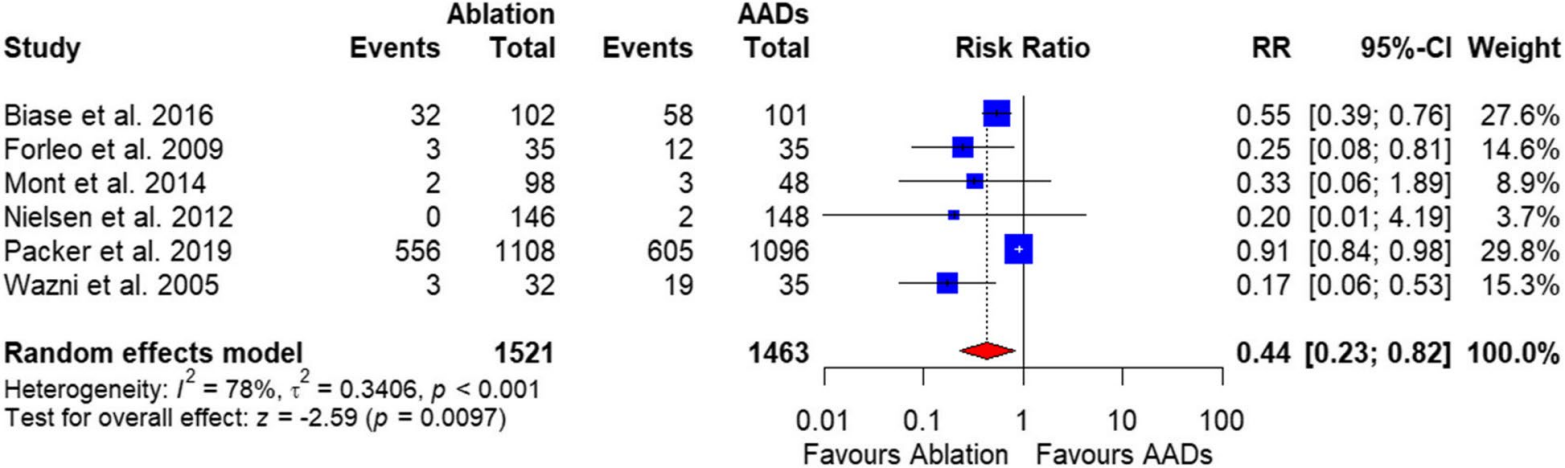
Forrest plot of rate of hospitalization

The pooled analysis of 5 studies with total 2781 patients showed a significant lower hospitalization rate in the catheter ablation group compared with AADs [RR = 0.44, 95%CI (0.23, 0.82), *P* = 0.009] (Fig. 5**).** The data were heterogenous (*P* = 0.001, I^2^ = 78%), and this was resolved by excluding Packer et al. 2019 (*P* = 0.93, I^2^ = 0). After resolving heterogeneity, the hospitalization rate in the catheter ablation group remained lower compared with AADs [RR = 0.34, 95%CI (0.18, 0.64), *P* ˂ 0.0001] (Supplementary Fig. 2).

##### Adverse events

**Fig. 6:**
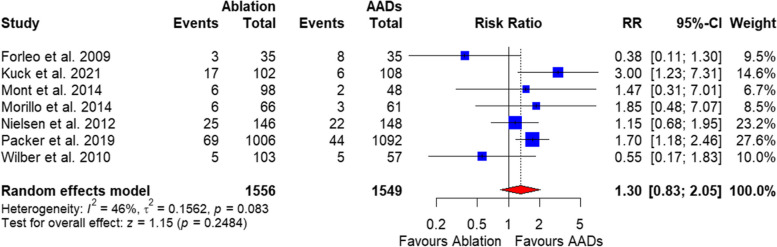
Forest plot of adverse events

Any adverse events rate in the catheter ablation group was comparable to AADs based on the pooled analysis of 7 studies with a total of 3105 patients [RR = 1.30, 95%CI (0.83, 2.05), *P* = 0.24], and the data were heterogenous (*P* = 0.08, I^2^ = 46%) (Fig. 6**)**, and this was resolved by excluding Forleo et al. 2019 (I^2^ = 23%). After resolving heterogeneity, the any AEs outcome in the catheter ablation group was significantly higher compared with AADs [RR = 1.51, 95%CI (1.08, 2.10), *P* = 0.02] (Supplementary Fig. 3).

**Fig. 7:**
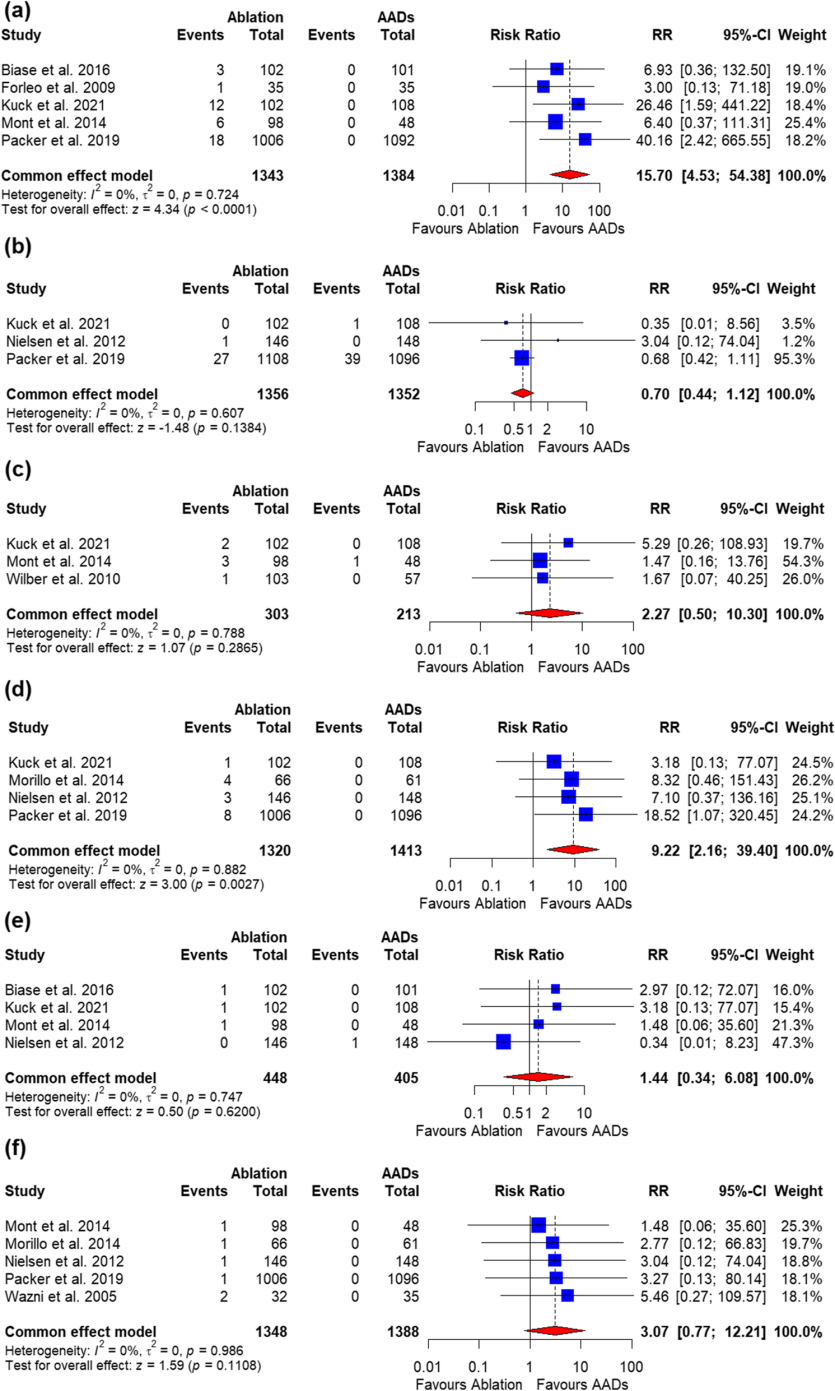
Forrest plot of death

The procedure-related AEs and cardiac tamponade in the catheter ablation group were significantly higher than those in the AADs group [RR = 15.70, 95%CI (4.53, 54.38), *P* < 0.0001] and [RR = 9.22, 95%CI (2.16, 39.40), *P* = 0.0027], respectively. The data were homogenous (*P* = 0.72, I2 = 0%) and heterogeneous (*P* = 0.88, I2 = 0%) (Fig. 7a and d). Stroke, vascular access complications, pericardial effusion, and pulmonary-vein stenosis were all similar between the two groups (Fig. 7b, c, d, e, and f, respectively). All the outcomes were homogenous.

##### All-cause mortality

**Fig. 8:**
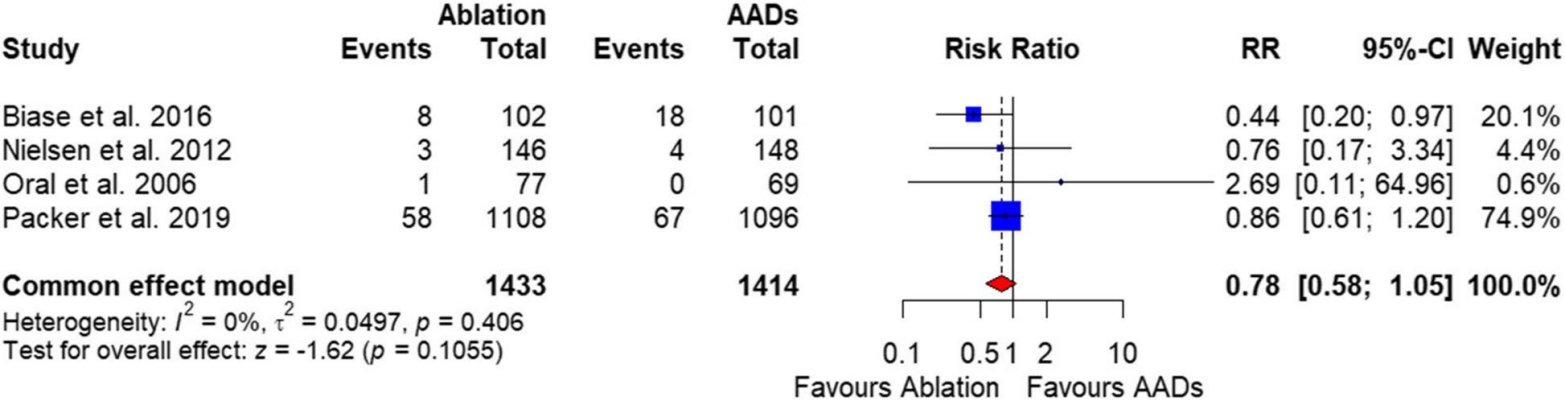
Forest plot of change in SF-36-Physical component

There was no significant difference between the two groups in the all-cause mortality rate [RR = 0.78, 95%CI (0.58, 1.05), *P* = 0.10], and the data were homogenous (*P* = 0.40, I^2^ = 0%) (Fig. 8).

#### Secondary outcomes

##### Quality of life

**Fig. 9:**
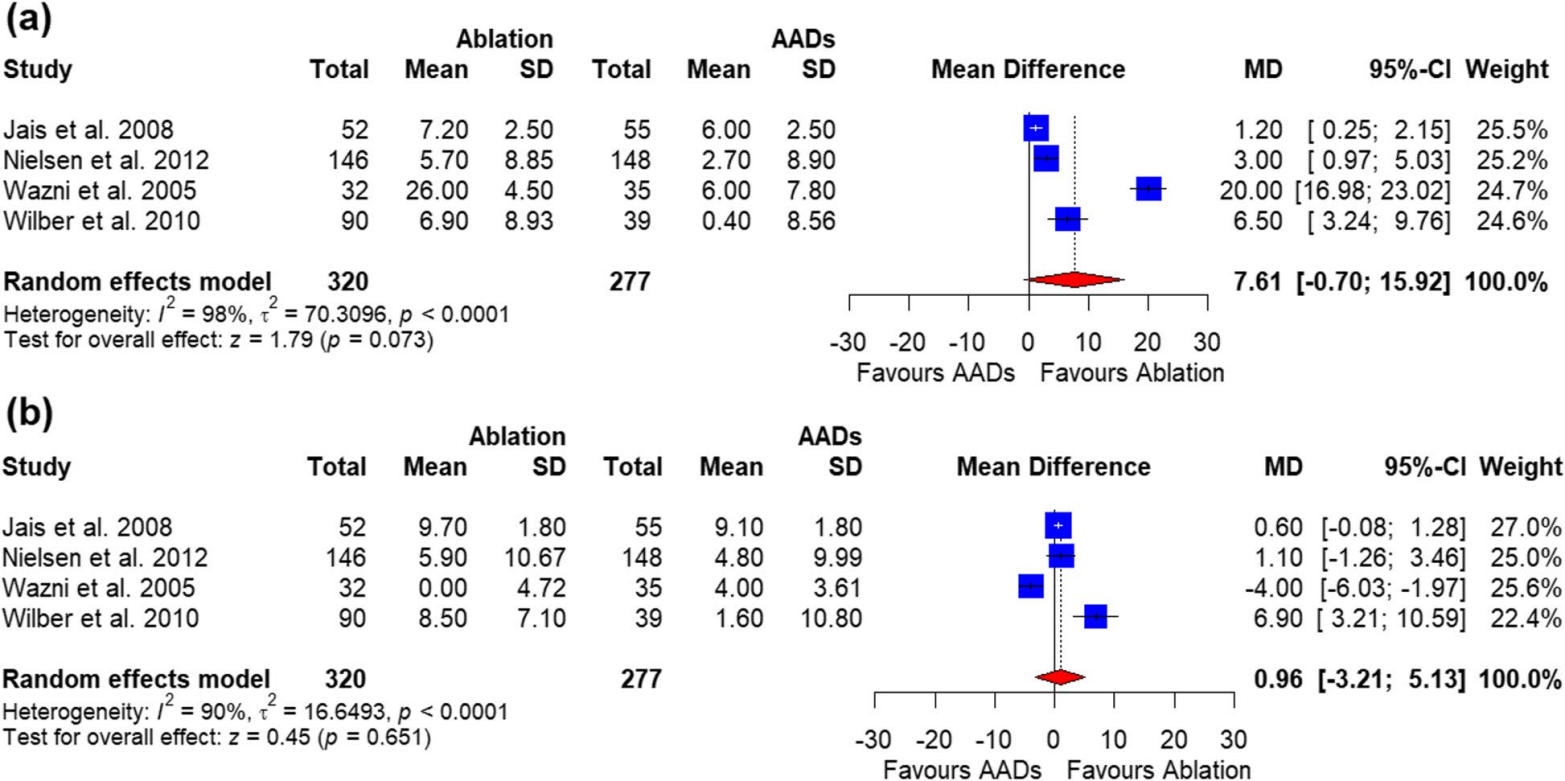
Forest plot of change in SF-36-Mental component

Our pooled analysis showed a significant improvement in the SF-36 physical component in the catheter ablation group compared with AADs [MD = 7.61, 95%CI (-0.70, 15.92), *P* = 0.07]. The data were heterogenous (*P* ˂ 0.0001, I^2^ = 98%) (Fig. 9a), and this heterogeneity could not be resolved with the sensitivity analysis (Supplementary Fig. 3). The pooled analysis of the 3 studies showed no significant difference between the catheter ablation group and AADs in the change in SF-36-Mental component [MD = 0.96, 95%CI (-3.21, 5.13), *P* = 0.65]. The data were heterogenous (*P* ˂ 0.0001, I^2^ = 90%) (Fig. 9b**)**, and this heterogeneity could not be resolved (Supplementary Fig. 4).

#### Subgroup analysis

Subgroup analysis based on the type of AF didn’t show a significant difference between those with persistent and paroxysmal AF regarding arrythmia recurrence; however, the heterogeneity was partially resolved (Supplementary Fig. 5).

Subgroup analysis based on the duration of follow-up showed that studies with a follow-up duration of 6–12 months were significantly higher than those with more than one year (*P* < 0.05); however, in both subgroups, ablation showed significant lower risk or AF recurrence (Supplementary Fig. 6).

#### Meta-regression

A meta-regression analysis revealed a statistically significant positive correlation between the duration of follow-up and the pooled estimate for both arrhythmia recurrence (*r* = 0.02, *P* = 0.018).

## Discussion

In this systematic review and meta-analysis of 12 RCTs including 3977 patients with paroxysmal or persistent atrial fibrillation, catheter ablation was associated with significantly higher free from atrial arrhythmia recurrence between 6 months post-procedure compared to antiarrhythmic drug therapy. The pooled analysis found a 44% relative risk reduction for AF recurrence with ablation. Additionally, rates of hospitalization were substantially lower with ablation. However, these improved efficacy outcomes came at the cost of an increased risk of procedural-related complications and cardiac tamponade. No difference in all-cause mortality was noted between the treatment strategies.

Catheter ablation also demonstrated benefits to quality of life based on SF-36 scores. The physical health composite score improved by approximately 5 points more with ablation than with medications. However, the difference in mental health component score change was not statistically significant between groups. This data warrants further trials with larger sample size.

Our efficacy findings confirm an advantage of ablation aligned with previous meta-analyses in paroxysmal AF patients [31–33]. However, few prior reviews assessed outcomes separately for persistent AF or incorporated the latest-generation ablation tools and techniques. Our study is among the first to pool trial data across both paroxysmal and persistent AF. It provides updated estimates following major evolutions in both pharmacological and ablative treatment options.

While ablation offered efficacy benefits, it did confer moderately higher risk of complications like vascular injury and pericardial effusions. Yet, the 1.5 times greater adverse event rate aligns with expectations for a complex, invasive procedure relative to oral medications. The spectrum of complications was generally manageable without excess mortality. Careful patient selection, operator experience, and performing procedures in higher volume centers can help mitigate procedural risks. Future technological advances may also enhance the safety profile of AF ablation [34].

There remains debate around the appropriate timing for ablation in AF management pathways for stroke prevention. Most patients in these trials continued anticoagulation per guidelines. The open question of whether successful ablation can allow stopping anticoagulants after a several-month blanking periods requires further study through longitudinal cohort follow-up [35].

An important limitation in assessing ablation’s definitive role relates to discrepancies in healthcare systems and reimbursement models internationally. Due to high upfront facility costs, ablation is estimated to have unfavorable short-term economic profiles compared to generic AADs in European nationalized systems [36, 37]. Yet models suggest cost equivalence or even superiority over longer horizons for ablation, considering savings from downstream cardiovascular care [36]. Value-based research is needed to clarify clinical and financial trade-offs globally.

This meta-analysis synthesized only RCT data, supporting internal validity and causality for the treatment effects. However, gaps remain in real-world, generalizable evidence. Participants enrolled in trials may not reflect heterogeneous AF populations or community practice patterns. There was heterogeneity in some pooled analyses, attributable to differences in ablation methods, antiarrhythmic drug choice/dosing, AF type, and monitoring protocols. Another aspect could be the difference in sample size, such as Packer et al. 2019, which created significant heterogeneity but had a significantly higher sample size (2,204). However, the main effects remained consistent after sensitivity analyses. More high-quality head-to-head trials are warranted, particularly focusing on persistent AF cohorts.

This systematic review substantiates an overall beneficial efficacy and safety profile for catheter ablation over antiarrhythmic drug therapy for rhythm control in patients with symptomatic paroxysmal or persistent AF refractory to at least one medication attempt. Subgroup differences based on arrhythmia persistence, patient comorbidities, or other modifiers remain less clear. While evaluating long-term outcomes and ideal timing for ablation are needed, our results support catheter ablation as a recommendable treatment option in patients failing initial antiarrhythmic medications. Ablation is likely underutilized currently and could be offered more widely rather than pursuing multiple unsuccessful drug trials in AF patients. Shared decision-making discussions should weigh upfront risks against reduced arrhythmia burden, hospitalizations, and improved quality of life after ablation. Additional study is justified to enhance patient selection criteria, procedural technique, management protocols and longitudinal monitoring to optimize the risk-benefit ratio with AF ablation.

### Supplementary Information

Supplementary Material 1.

## Data Availability

All data generated or analyzed during this study are included in this published article.
