# Supplementary material for: Antiarrhythmic drug therapy and catheter ablation in patients with paroxysmal or persistent atrial fibrillation: a systematic review and meta-analysis
Source: BMC Cardiovasc Disord. 2024 Jun 25;24:321. doi: 10.1186/s12872-024-03983-z (PMC11197351; doi:10.1186/s12872-024-03983-z)

Supplementary Figures:

Supplementary Fig 1: Leave one teat Recurrent Atrial Arrhythmia

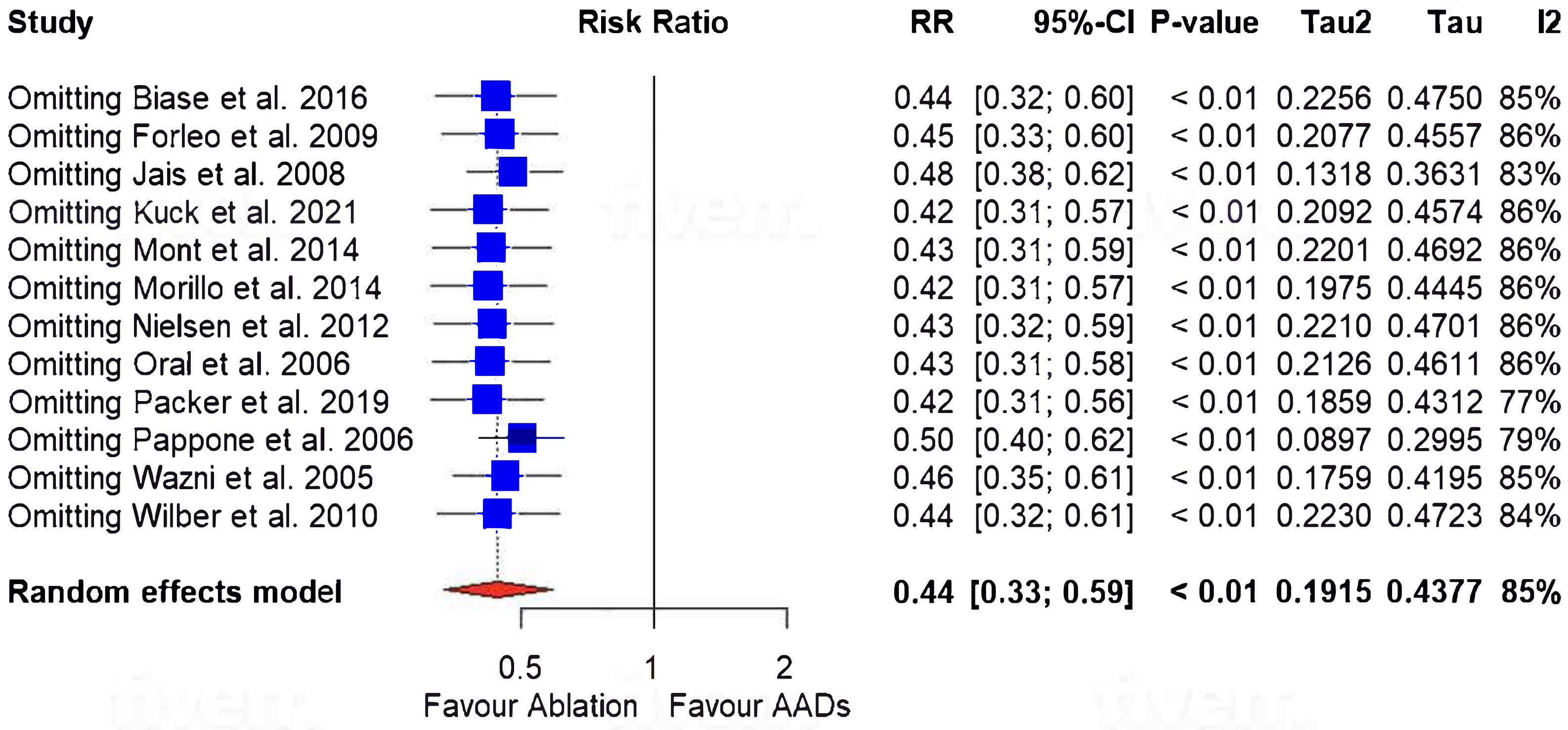

Supplementary Fig 2: Leave one teat Recurrent Hospitalization

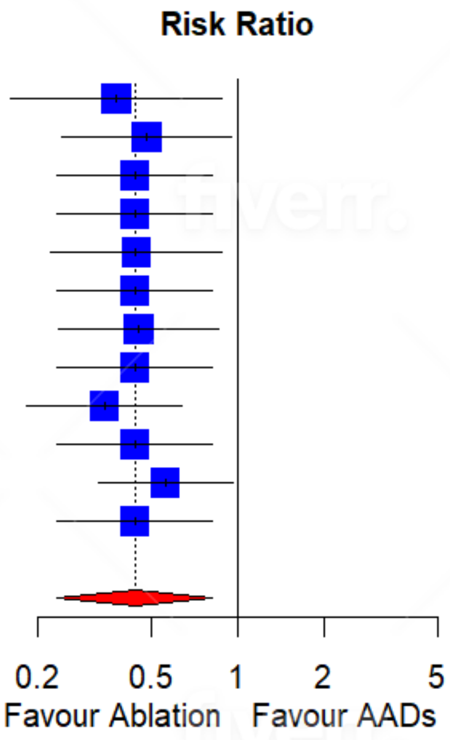

Study	RR	95%-CI	P-value	Tau2	Tau	I2
Omitting Biase et al. 2016	0.38	[0.16; 0.88]	0.02	0.5166	0.7188	74%
Omitting Forleo et al. 2009	0.48	[0.24; 0.95]	0.03	0.3442	0.5867	79%
Omitting Jais et al. 2008	0.44	[0.23; 0.82]	< 0.01	0.3406	0.5836	78%
Omitting Kuck et al. 2021	0.44	[0.23; 0.82]	< 0.01	0.3406	0.5836	78%
Omitting Mont et al. 2014	0.44	[0.22; 0.88]	0.02	0.3900	0.6245	82%
Omitting Morillo et al. 2014	0.44	[0.23; 0.82]	< 0.01	0.3406	0.5836	78%
Omitting Nielsen et al. 2012	0.45	[0.24; 0.86]	0.01	0.3476	0.5895	82%
Omitting Oral et al. 2006	0.44	[0.23; 0.82]	< 0.01	0.3406	0.5836	78%
Omitting Packer et al. 2019	0.34	[0.18; 0.64]	< 0.01	0.1892	0.4350	26%
Omitting Pappone et al. 2006	0.44	[0.23; 0.82]	< 0.01	0.3406	0.5836	78%
Omitting Wazni et al. 2005	0.56	[0.32; 0.97]	0.04	0.1912	0.4372	73%
Omitting Wilber et al. 2010	0.44	[0.23; 0.82]	< 0.01	0.3406	0.5836	78%
**Random effects model**	**0.44**	**[0.23; 0.82]**	**< 0.01**	**0.3406**	**0.5836**	**78%**

Supplementary Fig 3: Leave one test Change in SF-36-Physical Component

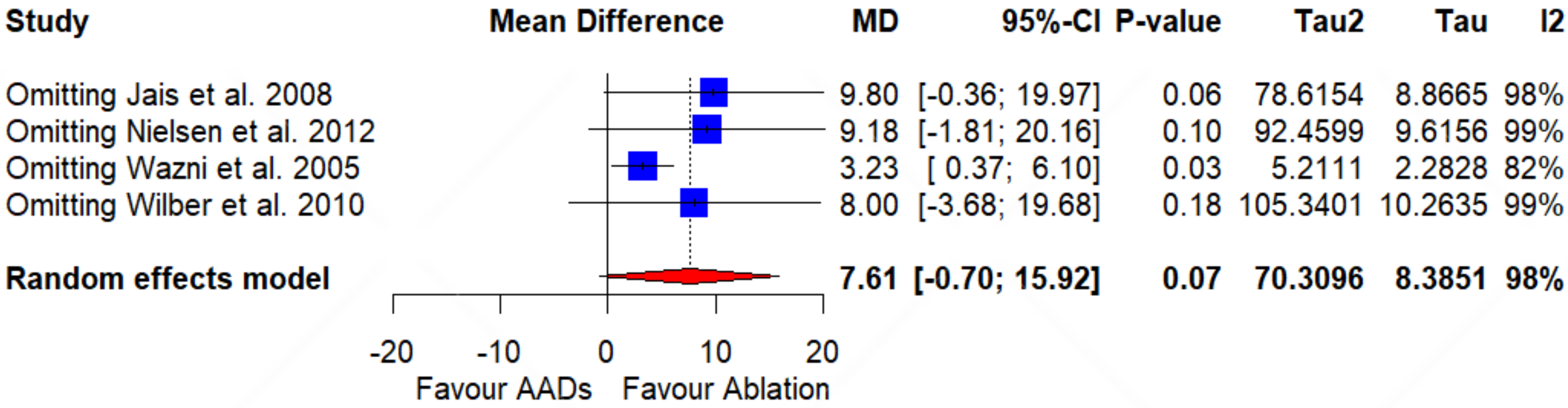

Supplementary Fig 4: Leave one test Change in SF-36 Mental Component

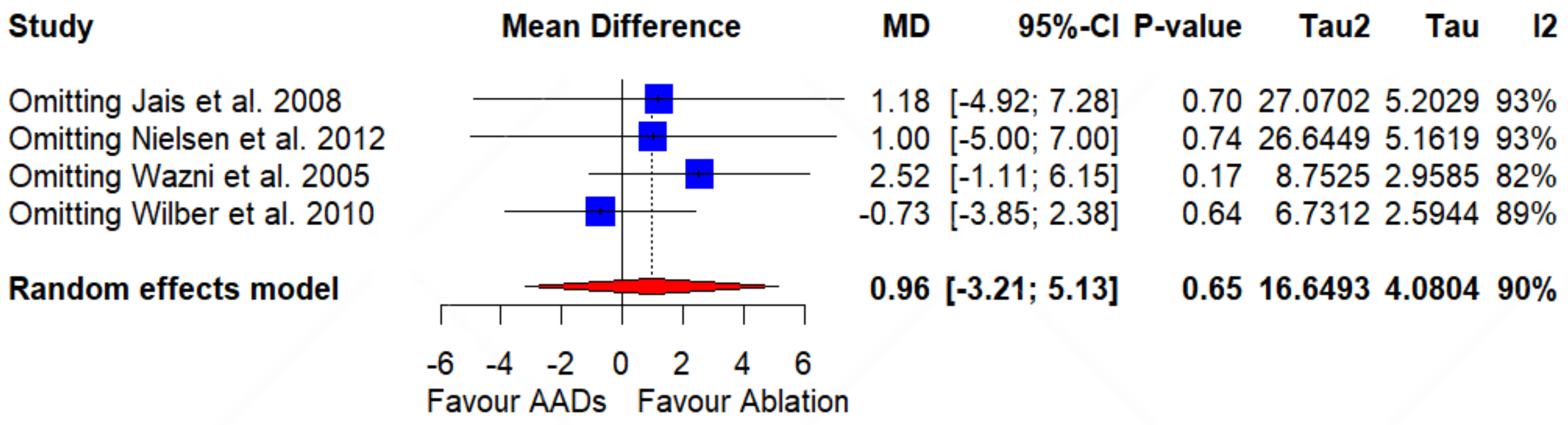

Supplementary Figure 5: Subgroup Analysis of Persistent AF and Paroxysmal AF

Study	Ablation Events	Ablation Total	AADs Events	AADs Total	RR	95%-CI	Weight
**Persistent AF**							
Biase et al. 2016	31	102	67	101	0.46	[0.33; 0.63]	9.4%
Mont et al. 2014	39	98	34	48	0.56	[0.41; 0.76]	9.5%
Oral et al. 2006	20	77	29	69	0.62	[0.39; 0.99]	8.3%
**Random effects model**		**277**		**218**	**0.53**	**[0.43; 0.65]**	**27.2%**
Heterogeneity: $I^2 = 0\%$, $\tau^2 = 0$, $p = 0.515$							
Test for effect in subgroup: $z = -6.22$ ($p < 0.0001$)							
**Both types**							
Forleo et al. 2009	7	35	20	35	0.35	[0.17; 0.72]	6.3%
Packer et al. 2019	554	1108	756	1096	0.72	[0.68; 0.78]	10.6%
**Random effects model**		**1143**		**1131**	**0.55**	**[0.28; 1.10]**	**16.9%**
Heterogeneity: $I^2 = 74\%$, $\tau^2 = 0.1965$, $p = 0.049$							
Test for effect in subgroup: $z = -1.69$ ($p = 0.0919$)							
**Paroxysmal AF**							
Jais et al. 2008	6	52	42	55	0.15	[0.07; 0.33]	6.0%
Kuck et al. 2021	69	128	108	127	0.63	[0.53; 0.76]	10.3%
Morillo et al. 2014	27	66	35	61	0.71	[0.50; 1.02]	9.1%
Nielsen et al. 2012	22	146	43	148	0.52	[0.33; 0.82]	8.3%
Pappone et al. 2006	13	99	75	99	0.17	[0.10; 0.29]	7.8%
Wazni et al. 2005	4	32	22	35	0.20	[0.08; 0.51]	4.8%
Wilber et al. 2010	35	103	47	56	0.40	[0.30; 0.54]	9.6%
**Random effects model**		**626**		**581**	**0.36**	**[0.23; 0.58]**	**55.9%**
Heterogeneity: $I^2 = 86\%$, $\tau^2 = 0.3308$, $p < 0.001$							
Test for effect in subgroup: $z = -4.23$ ($p < 0.0001$)							
**Random effects model**		**2046**		**1930**	**0.44**	**[0.33; 0.59]**	**100.0%**
Heterogeneity: $I^2 = 85\%$, $\tau^2 = 0.1915$, $p < 0.001$							
Test for overall effect: $z = -5.69$ ($p < 0.0001$)							
Test for subgroup differences: $\chi^2_2 = 2.16$, df = 2 ($p = 0.339$)							

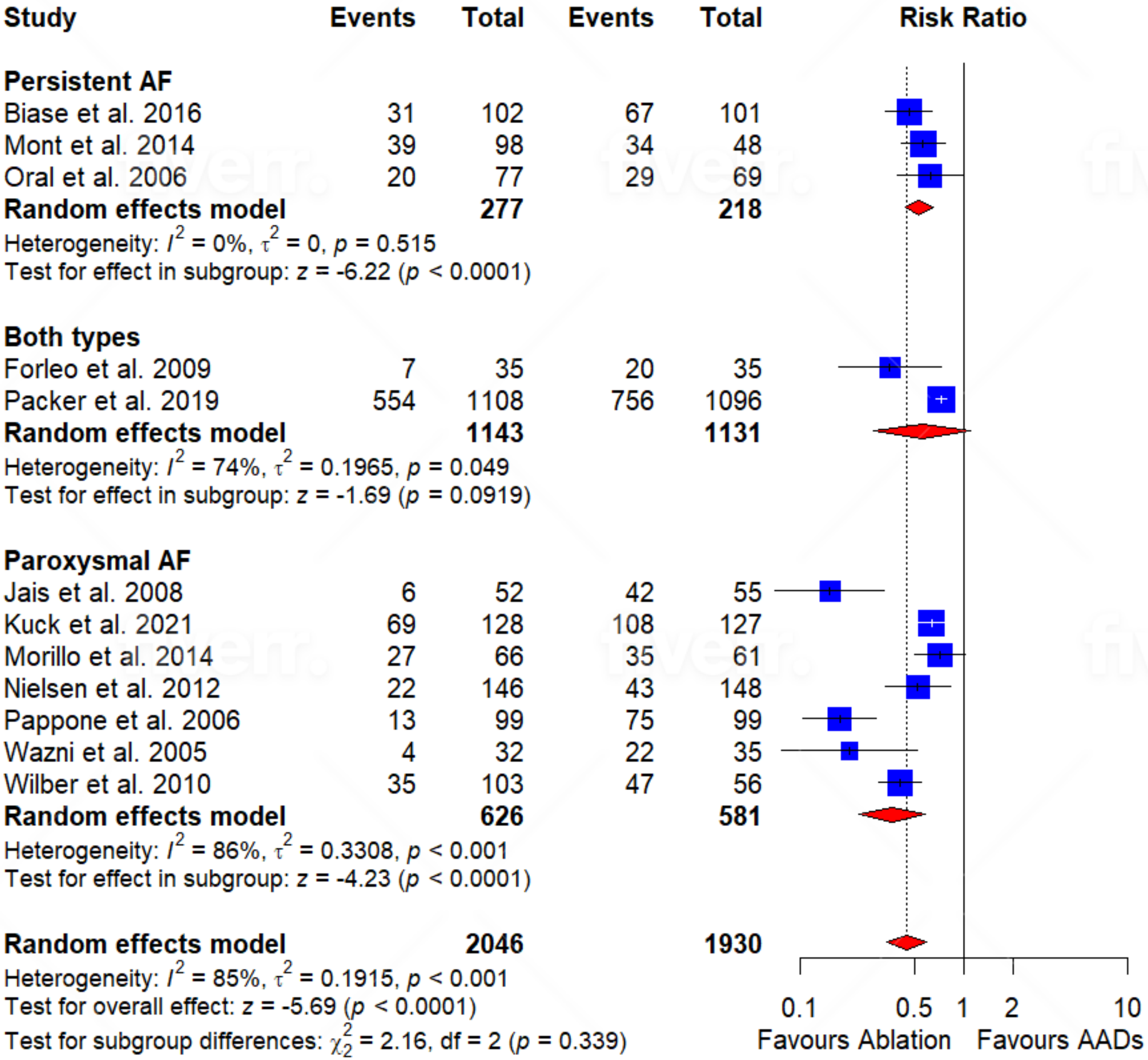

Supplementary Figure 6: Subgroup Analysis of Followup of 6-12 months and >12 months

Study	Ablation Events	Ablation Total	AADs Events	AADs Total	RR	95%-CI	Weight
**> 12 months**							
Biase et al. 2016	31	102	67	101	0.46	[0.33; 0.63]	9.4%
Kuck et al. 2021	69	128	108	127	0.63	[0.53; 0.76]	10.3%
Morillo et al. 2014	27	66	35	61	0.71	[0.50; 1.02]	9.1%
Nielsen et al. 2012	22	146	43	148	0.52	[0.33; 0.82]	8.3%
Packer et al. 2019	554	1108	756	1096	0.72	[0.68; 0.78]	10.6%
**Random effects model**		**1550**		**1533**	**0.63**	**[0.53; 0.74]**	**47.7%**
Heterogeneity: $I^2 = 61\%$, $\tau^2 = 0.0194$, $p = 0.036$							
Test for effect in subgroup: $z = -5.44$ ($p < 0.0001$)							
**6-12 months**							
Forleo et al. 2009	7	35	20	35	0.35	[0.17; 0.72]	6.3%
Jais et al. 2008	6	52	42	55	0.15	[0.07; 0.33]	6.0%
Mont et al. 2014	39	98	34	48	0.56	[0.41; 0.76]	9.5%
Oral et al. 2006	20	77	29	69	0.62	[0.39; 0.99]	8.3%
Pappone et al. 2006	13	99	75	99	0.17	[0.10; 0.29]	7.8%
Wazni et al. 2005	4	32	22	35	0.20	[0.08; 0.51]	4.8%
Wilber et al. 2010	35	103	47	56	0.40	[0.30; 0.54]	9.6%
**Random effects model**		**496**		**397**	**0.33**	**[0.21; 0.50]**	**52.3%**
Heterogeneity: $I^2 = 77\%$, $\tau^2 = 0.2447$, $p < 0.001$							
Test for effect in subgroup: $z = -5.12$ ($p < 0.0001$)							
**Random effects model**		**2046**		**1930**	**0.44**	**[0.33; 0.59]**	**100.0%**

Heterogeneity: $I^2 = 85\%$, $\tau^2 = 0.1915$, $p < 0.001$
Test for overall effect: $z = -5.69$ ($p < 0.0001$)
Test for subgroup differences: $\chi^2_1 = 7.76$, df = 1 ($p = 0.005$)

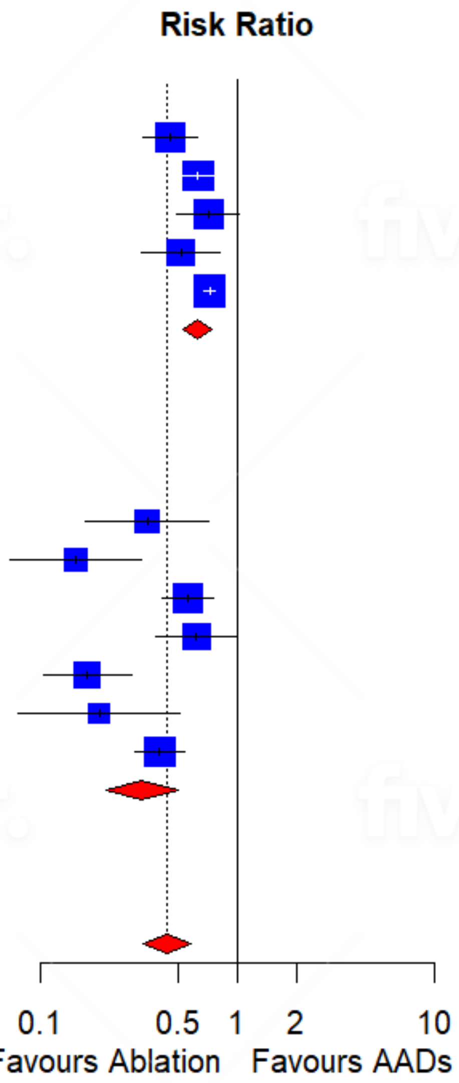

Supplement: Supplementary file 1 — Supplementary Material 1. [file 12872_2024_3983_MOESM1_ESM.pdf]
